# Solid Dispersion Pellets: An Efficient Pharmaceutical Approach to Enrich the Solubility and Dissolution Rate of Deferasirox

**DOI:** 10.1155/2020/8583540

**Published:** 2020-06-23

**Authors:** Ali Farmoudeh, Anahita Rezaeiroshan, Mohammadreza Abbaspour, Ali Nokhodchi, Pedram Ebrahimnejad

**Affiliations:** ^1^Department of Pharmaceutics, Faculty of Pharmacy, Mazandaran University of Medical Sciences, Sari, Iran; ^2^Student Research Committee, Faculty of Pharmacy, Mazandaran University of Medical Sciences, Sari, Iran; ^3^Targeted Drug Delivery Research Center, Pharmaceutical Technology Institute, Mashhad University of Medical Sciences, Mashhad, Iran; ^4^Pharmaceutics Research Lab, School of Life Sciences, University of Sussex, Brighton, UK; ^5^Pharmaceutical Science Research Center, Hemoglobinopathy Institute, Mazandaran University of Medical Sciences, Sari, Iran

## Abstract

Deferasirox (DFX) is an oral iron-chelating agent and classified into class II of the Biopharmaceutics Classification System. Low bioavailability of the drug due to insufficient solubility in physiological fluids is the main drawback of DFX. The idea of the current study was to explore the potential of solid dispersion (SD) as an effective method to improve the dissolution rate of DFX in pellets. The SDs were made by the solvent evaporation technique using polyethylene glycol 4000 (PEG 4000) and polyvinylpyrrolidone K25 with different drug-to-carrier ratios. Then, the dispersion was milled and mixed with other components and the mixture layered on sugar-based cores by pan coating technique. The pellets were evaluated in terms of size distribution, morphology (SEM), and dissolution behaviour. Drug-polymer interactions were studied using differential scanning calorimetry (DSC), X-ray diffraction study (XRD), and Fourier transformation infrared (FTIR) spectroscopy. The pellets coated with SD showed a remarkable rise in the solubility of DFX than that of free drug-loaded pellets. The dispersion with PVP K25 showed a faster dissolution rate as compared to other mixtures. The DSC and XRD analysis indicated that the drug was in the amorphous state when dispersed in the polymer. The FTIR studies demonstrated any ruled out interaction between drug and polymer. The SEM showed smoothness on the surface of the pellets. It is resolved that the SD method considerably enriched the dissolution rate of DFX in pellets, which can also be utilized for other poorly water-soluble drugs.

## 1. Introduction

Oral administration is still the most accessible and favorite way of drug delivery among patients because of its simple application. This interest led researchers to focus on enhancing the dissolution of low-soluble drugs for oral delivery through an enhancement in solubility and control of drug release, making the formulations more biocompatible, and improving bioavailability. The solubility of molecules in the biological fluid is the major and essential process for the intestinal absorption of drug compounds. One of the main reasons for low absorption and high individual variability of drugs is the lack of solubility in physiological fluids. Solubility is one of the critical limitations in drug formulation and biopharmaceutical pattern [1]. Approximately 40% of novel drugs discovered by pharmaceutical corporations have difficulties in aqueous solubility, and many formulations have failed because of insufficient biopharmaceutical properties [2, 3].

Recently, there has been a great trend and major attention in pharmaceutical research to the ways of enriching the dissolution percentage of low-soluble molecules [4, 5]. Various strategies such as micro- and nanosizing, micelle formation, salt formation, complexation, liquisolids, liqui-pellets, and solid dispersion (SD) method have been exploited to enhance the dissolution rate and consequently improve the absorption of low-soluble agents at the gastrointestinal (GI) tract [6–8].

In 1961, Sekiguchi and Obi introduced a new approach for enhancing the dissolution of low water-soluble active pharmaceutical ingredients (APIs) known as SD technique [9]. In this technique, the conversion of particles from crystals to amorphous forms and size reduction to molecular dimensions thereby enhancing the particle surface area and forming hydrogen bonds between active agents and hydrophilic carriers are key mechanisms to elevating the solubility of low water-soluble molecules [2, 10]. The solubility of insoluble APIs is increased by dispersing them in a water-soluble polymer [11]. Polyoxyethylene glycol (PEG) and polyvinyl pyrrolidone (PVP) have a special hydrophilic character and are widely used in SD techniques. [12, 13]. In addition, these polymers are biocompatible and biodegradable with high safety and low cost which make them be good selections for this purpose. Recently, fabrication of multiparticulate dosage forms has been considered an encouraging strategy to elevating the dissolution rate of class II drugs through enhancement in the surface area [14–18]. Among oral dosage forms, pellets offer several benefits; they move freely through the GI tract and thus cause less irritation and better distribution and improve drug absorption; they can reduce dose dumping, which results in fewer adverse effects and plasma fluctuation [5, 16, 19]. Pellets are defined as spherical agglomerations fabricated using various pelletization methods including extrusion/spheronization, spray drying, and powder layering techniques. In the pelletization techniques, the fine powders are aggregated to form larger, spherical particles called pellets [20]. Pellets have an enormous surface area in contrast to tablets and capsules, which increases their exposure and interaction with the surrounding medium, thereby increasing the dissolution rate of the drug [21–24]. Deferasirox (DFX) is an iron chelator drug that helps to remove excess iron in the body, and it is usually administered in the treatment of beta-thalassemia and sickle cell disorders [25]. Recently, DFX has shown anticancer characteristics against various cancer cell lines [6, 14]. DFX pertains to the Biopharmaceutics Classification System (BCS) class II compounds that are practically insoluble in water [14, 15, 25]. Banerjee reported a solubility of 0.038 mg/ml for DFX in the aqueous medium at 37°C [26]. So this drug is poorly absorbed by oral administration [6]. Generally, the traditional SD technology produces a bulky powder stick together, which does not flow well, and the obtained materials should be subjected to milling to get the desired particle size. In addition, longer milling can cause issues such as changes in the polymorphic form. To the best of our knowledge, there is no report using the SD method to increase the solubility of DFX by pellet formulation. The current idea of using a combination of solid dispersion and pelletisation technology not only can enhance the dissolution rate but also overcomes the flow properties and issues associated with milling (as no milling is needed). In this study, the effect of hydrophilic polymers (PEG 4000 and PVP K-25) on the solubility of DFX was investigated. The drug-polymer SD was prepared by the solvent evaporation technique, and the dispersion was loaded into neutral pellets by powder layering. The *in vitro* release was performed, and the kinetics of the DFX release from the pellets were studied.

## 2. Materials and Methods

### 2.1. Materials

DFX was obtained from Osvah Pharmaceutical Company (Tehran, Iran). PEG4000, PVP K-25, potassium dihydrogen phosphate, sodium hydroxide (NaOH), and all HPLC grade solvents were obtained from Merck KGaA (Darmstadt, Germany). Ultrapure deionized water was prepared by the Human Ultra-Pure System (Human Corp, Korea).

### 2.2. Preparation of SDs

SDs of DFX in PEG4000 or PVP K25 containing different weight ratios (Table 1) were fabricated by the solvent evaporation technique [27, 28]. The polymer was dissolved in 10 ml of water, and the drug was dissolved in the same volume of ethanol. The polymer solution was added to the stirring drug solution to make it uniform. The solvents were removed under vacuum at 45°C, and the resulting residue was freeze-dried to remove the remaining water. The dried materials were crushed using mortar and pestle and passed through a 170-mesh screen.

### 2.3. Phase-Solubility Analysis

The determination of solubility of free DFX, physical mixtures of drugs and polymers, and SD were performed by adding an additional amount of each sample in conical flasks containing 50 ml of deionized water. The suspension formed was equilibrated under continuous stirring for 24 hours at 37°C and then passed through a 0.22 *μ*m membrane filter to make a clear solution for analysis.

### 2.4. Preparation of Pellets

To prepare pellets with a smooth surface, the drug and excipients were screened through a 170-mesh sieve prior to mixing. According to Table 2, drug-polymer SDs and excipients were weighed and blended uniformly. Then, the mixture was coated on the inert core pellets by powder-layering technique and using a conventional coating pan model DKE/DKS (Erweka, Heusenstamm, Germany). The binding solution (PVP 0.5%) and the drug-excipient mixture were sprayed consecutively onto the null pellets at a constant rate to increase the size of pellets [20–22].

### 2.5. Sieve Analysis

The size distribution was determined using 50 g pellet samples. To this end, an Erweka vibration sieve (Erweka, Germany) through a nest of sieves using 14-35 mesh screens was used and 100 grams of pellets was shaken for 5 minutes [29]. The amount of materials left on each sieve was weighed, and particle size distribution was constructed.

### 2.6. Reversed-Phase HPLC Analysis

The DFX concentrations of different formulations were determined by HPLC (KNAUER D-14163; Berlin, Germany). Chromatographic separations were performed using a KNAUER C18 column (4.6 mm × 250 mm), UV detector set at 245 nm, and ChromGate Clint software version 3.1.7. The mobile phase consisted of acetonitrile : methanol : water (40 : 20 : 40), the volume of injection was 20 *μ*l, and the flow rate was 0.7 ml/min. The standard curve for DFX was constructed over a range of 0.5–50 *μ*g/ml [24, 30].

### 2.7. Determination of Drug Content and Entrapment Efficiency

The number of pellets equivalent to 100 mg of DFX was crushed, and their powder was dissolved in 100 ml of ethanol and diluted with HPLC mobile phase to produce 30 *μ*g/ml solution. Then, the solution was passed using a 0.22 *μ*m filter and measured using the HPLC-UV system to analyze the amount of DFX in pellets. The entrapment efficiency was also obtained by the following equation:

(1) $$\begin{array}{c} \text{Drug entrapment efficiency }\left(\%\right)=\left(\frac{\text{AQ}}{\text{TQ}}\right)\times 100, \end{array}$$

where AQ is the actual quantity of drug and TQ is the 100% theoretical quantity of drug present in the surface of pellets (i.e., initial loading dose).

### 2.8. Drug Dissolution

Dissolution was tested in a USP type I (basket) dissolution tester. Pellets (equivalent to 20 mg of DFX) were put into the Basket and were rotated at 100 rpm in phosphate buffer (pH 6.8, at 37 ± 0.5°C) as a dissolution medium. At predetermined time intervals, 2 ml of medium was withdrawn and filtrated and diluted with the mobile phase (1/5) and analyzed by the HPLC as described above.

The dissolution efficiency (DE) of the samples was determined based on the area under the dissolution curve between the selected time points (*t*_1_ and *t*_2_) which is the percentage of the curve at maximum dissolution (y100) over the same time. This concept was suggested by Khan in 1975 [31] and is calculated by the following equation:

(2) $$\begin{array}{c} \text{Dissolution efficiency }\left(\text{DE}\right)=\frac{\int_{t_{1}}^{t_{2}}y\cdot dt}{y100\cdot \left(t_{2}-t_{1}\right)}\times 100. \end{array}$$

In this study, DE from 0 to 10 or 60 min (expressed as %DE10 and %DE60, respectively) was calculated using the trapezoidal method.

### 2.9. Drug Release Kinetics

Four mathematical kinetic models namely zero-order, first-order, Korsmeyer-Peppas, and Higuchi which were employed to identify the mechanism of drug release from SD pellets. The best model was identified on the basis of the determination coefficient (*r*^2^) for each model. After 180 minutes, most of the drug was released from the samples and the drug release rate in all formulations decreased sharply, so the percentage of drug released up to180 minutes was considered in the kinetic models.

### 2.10. Differential Scanning Calorimetry (DSC)

Samples of free DFX, powdered drug-polymer SDs, and physical mixtures were transferred into aluminium pans (5 mg), and the pans were sealed. DSC analysis was conducted by a PerkinElmer DSC model pyris6 (PerkinElmer, Norwalk, USA). DSC runs were performed from 30 to 300°C at the 10°C/min heating rate. An empty aluminium pan was utilized as a reference material to calibrate the DSC temperature scale and enthalpic response [32]. Nitrogen was used as a purge gas, flowing through the apparatus at 20 cm^3^min^−1^.

### 2.11. Fourier Transform Infrared (FTIR)

DFX, polymers, physical mixtures, and SDs were evaluated by FTIR. Specimens of formulations were dispersed in KBr and compressed into transparent tablets. Then, the tablets were exposed to FTIR recording on the FTIR-One spectrometer (PerkinElmer, Norwalk, USA). The scanning range was 4000–450 cm^−1^, and the resolution was 1 cm^−1^.

### 2.12. Scanning Electron Microscopy (SEM)

The morphology of free DFX, polymers, SDs, and pellet surface was investigated using SEM (model FEI Quanta 200, FEI Company, USA) with a resolution of 3.0 nm. The specimens were primarily coated with a thin gold layer before the investigation to create electrical conductivity (at 30 kV).

### 2.13. X-Ray Powder Diffraction (XRPD)

XRPD of specimens was performed by an X-ray diffractometer model D8-Advance (Bruker AXS, Karlsruhe Germany). Measurement conditions included target Cu Ka radiation at 40 kV and 30 mA. The specimens were analyzed in the 2*θ* angle range of 4–45° at a scanning speed of 10° min^−1^.

### 2.14. Statistics

Data were reported as the mean ± standard deviation (SD) of three determinations. Comparison among groups was carried out by one-way analysis of variance (one-way ANOVA) followed by Tukey's multiple comparisons employing SPSS 22 for Windows (SPSS, Chicago, IL). *p* value of less than 0.05 was accounted for statistically significant in all tests.

## 3. Results and Discussion

### 3.1. Phase-Solubility Analysis

The phase solubility studies on DFX and its SD preparations with PVP or PEG were carried out. Aqueous solubility of DFX was observed to be 38.02 ± 0.08 *μ*g/ml (Table 1), indicating DFX as a practically insoluble drug. Phase solubility indicated that the solubility of DFX enhanced as a consequence of the concentration of polymers added to the formulation. Table 1 also shows that PVP K25 was more effective than PEG 4000 in the solubility enhancement of DFX (*p* < 0.05).

### 3.2. Sieve Analysis

**Figure 1:**
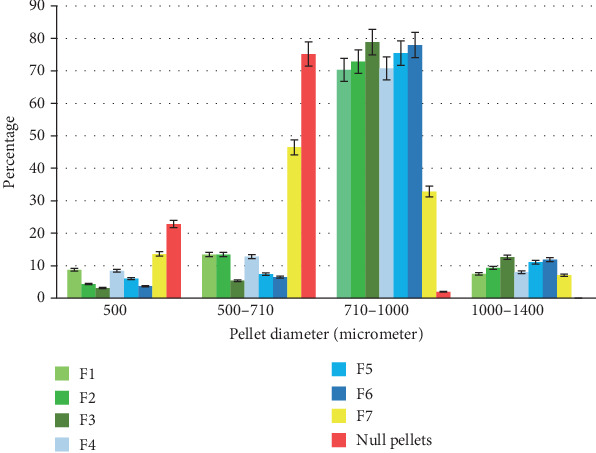
SD-coated and null pellets size distribution (data shown as the mean ± standard deviation, *n* = 3). For the details of the composition for each formulation, refer to Table 2.

The particle size distribution for all formulations is shown in Figure 1. The results showed that the majority (70 – 78.8%) of the pellets coated with SDs were ranged from 710 to 1000 *μ*m. So this size fraction was selected for further investigation.

### 3.3. Drug Content

**Figure 2:**
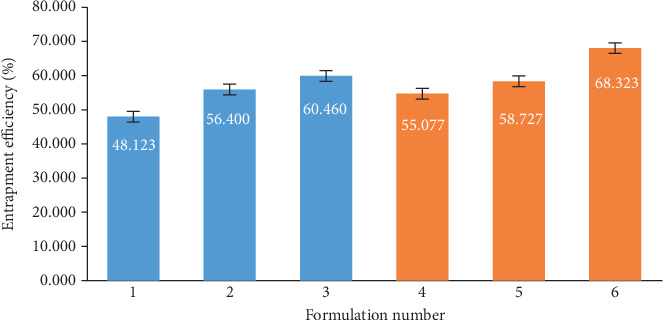
SD-coated pellets drug entrapment efficiency percent (data shown as the mean ± standard deviation, *n* = 3). For the details of the composition for each formulation, refer to Table 2.

**Figure 3:**
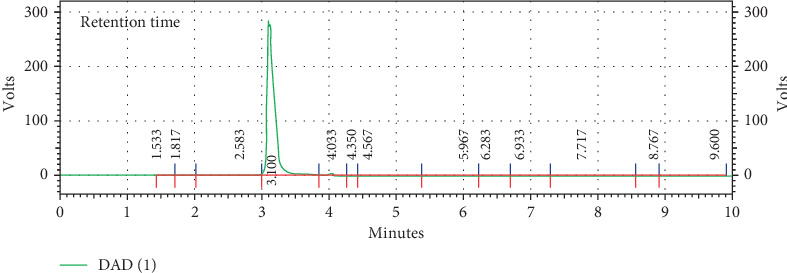
Typical HPLC chromatogram of DFX assay in SD-coated pellets.

The HPLC was used to determine DFX in the pellets. The standard curve for DFX was considered over a range of 0.5–50 *μ*g/ml and indicated to be linear (*y* = 155056*x* + 155322, *R*^2^ = 0.999). The results in Figure 2 showed that drug/PVP SD has a higher E.E.% than drug/PEG SD. PVP can have more adhesion property than PEG; therefore, this could be the main reason for the increased drug loading at the pellet surface in the case of PVP formulations [33]. A representative chromatogram obtained following the assay of DFX pellets is depicted in Figure 3.

### 3.4. Drug Dissolution Studies

**Figure 4:**
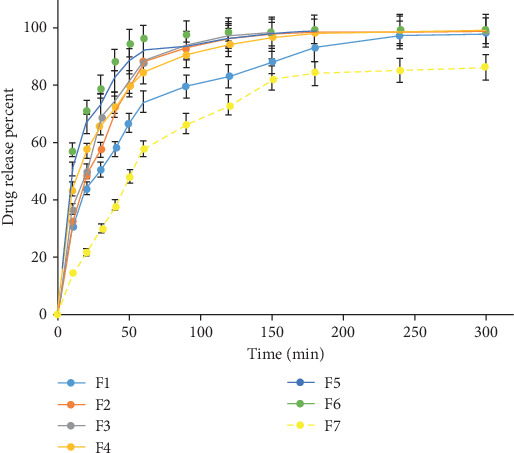
Dissolution profiles of pellet formulations (data shown as the mean ± standard deviation, *n* = 3). For the details of the composition for each formulation, refer to Table 2.

The solubility of a BCS Class II drug in the GI tract is the main limiting factor, and this, in turn, can reduce the bioavailability of this class of drugs. Consequently, it is high priority to raise the dissolution/solubility of DFX in the dissolution medium. The release profiles of free DFX and different SD formulations are illustrated in Figure 4. The dissolution efficiency (DE) after 10 and 60 min, as well as the percentage of DFX dissolved (DP) at the same time, is shown in Table 3. After 10 minutes, SDs showed significantly increased DP and DE compared to the free drug (*p* < 0.05). Similar results were observed after 60 minutes (*p* < 0.05). The abrupt and fast release of DFX from SDs can be related to its molecular dispersion in the polymeric carriers. In general, it is expected that in the SD system, a decrease in particle size should lead to further dissolution [34]. Moreover, drug wettability can be improved by drug-carrier hydrogen bonding [35]. The data for all solid dispersion formulations showed the best fit to the Korsmeyer-Peppas release model compared to other models shown in Table 4. Free drug-loaded pellets (formulation F7) showed good fitting to Higuchi and zero-order models. Previous studies have shown that drug release from controlled drug delivery dosage forms follows the Higuchi model [36]. In addition, the results of release were well fitted to the zero-order kinetics, indicating that in the F7 formulation the drug was released slowly at a constant rate. In the first three formulations (F1-F3), the release exponent n of the Peppas model was between 0.43 and 0.85, which shows a non-Fickian release mechanism, involving both diffusion and polymer erosion. In the next three formulations (F3-F6), the value of *n* calculated was found to be lower than 0.43, showing that the main drug release mechanism was diffusion (Fickian pattern) [37]. As in formulations F3-F6, PVP can act as a good binder for the formation of SD, which can hold the powders strongly in the pellet; therefore, erosion was not the dominant mechanism of drug release in these three formulations.

### 3.5. DSC

**Figure 5:**
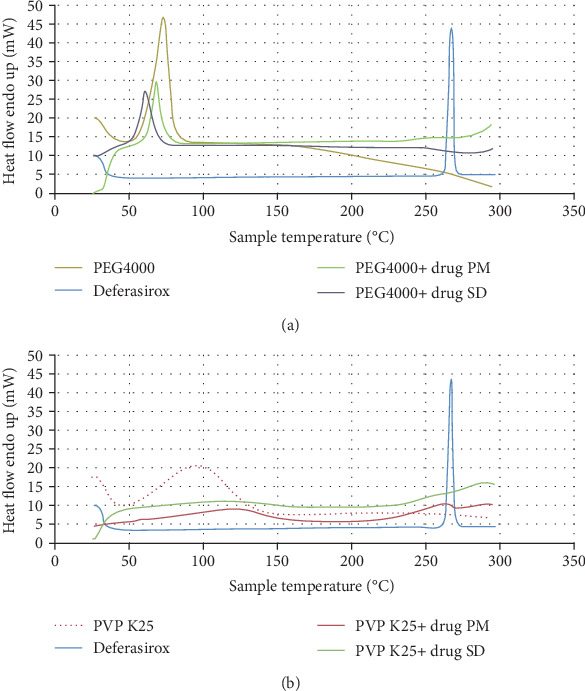
Differential scanning calorimetric curves of (a) DFX, PEG4000, DFX/PEG SD, and physical mixture (PM). (b) DFX, PVP K25, DFX-PVP SD, and physical mixture.

DSC was employed to assess the crystalline state of the active molecules and polymers in SDs. DSC traces of free DFX, PEG4000, PVP K25, DFX/polymer physical mixture, and SDs are illustrated in Figure 5. The free DFX displayed a single, sharp melting endothermic peak at 267.32°C confirming the drug is in its crystallinity state [38]. PEG was represented by a sharp endothermic pick at 58°C, and PVP showed a broad endotherm between 50 and 130°C, which represents the evaporation of water because of the hygroscopic characteristics of this polymer [39]. Physical mixtures of DFX and the polymers exhibited both endothermic transitions expressing the melting of drug and polymer, which ruled out any interaction between the drug and the polymers. The absence of the melting peak for the drug in the DSC thermogram of SDs indicates the drug in these samples is in an amorphous state or molecularly dispersed in the polymer.

### 3.6. FTIR Spectroscopic Analysis

**Figure 6:**
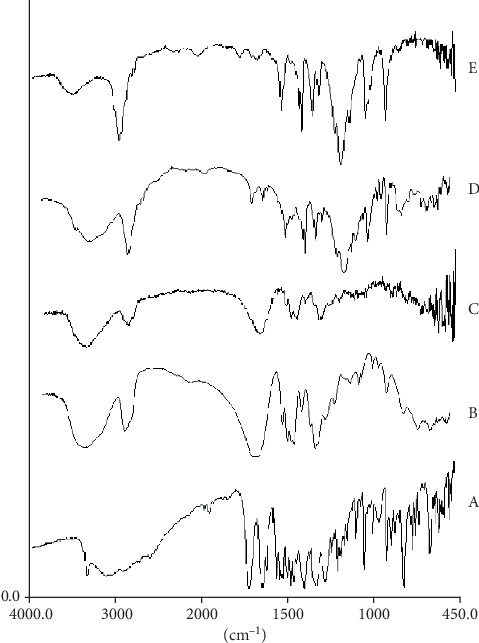
FTIR spectra of (a) free DFX, (b) PVP K25, (c) SD of DFX/PVP K25, (d) PEG4000, and (e) SD of DFX/PEG.

The FTIR spectra of DFX are illustrated in Figure 6. The characteristic peaks on the spectrum of free DFX were assigned as follows: 3318 cm^–1^ (O-H stretching), 3080 cm^−1^ (aliphatic thiazolidine stretching), 1680 cm^–1^ (acid, conjugated C=O stretching), 1608 cm^–1^ (C=N stretching), 1584.38 cm^−1^ (aromatic, C=C stretching), and 1352.06 cm^–1^ (O-H stretching of aromatic ring) [40]. The characteristic peaks on the spectrum of PVP K25 were assigned as follows: 2957 cm^–1^ (C-H stretching) and 1654 cm^–1^ (C=O stretching), and the very broadband at 3460 cm^–1^ was attributed to the presence of water. The PEG 4000 spectrum showed the main peaks at 3384 cm^–1^ (O-H stretching) and 1110 cm^–1^ (ether, C-O stretching). In SD of DFX and PVP, the absence of the hydroxyl peak of the drug and a significant decrease in the carbonyl group of PVP refers to the creation of hydrogen bonds between the hydroxyl group of DFX and the carbonyl group on the PVP pyrrole ring. PVP is able to form a hydrogen bond due to having a carbonyl group on its structure [41]. In the DFX/PEG SD spectrum, the carboxyl peak of DFX and the hydroxyl peak characteristics of PEG disappeared. These changes suggest the creation of hydrogen bonds between the DFX and the polymer. Similar studies on SDs of valdecoxib and temazepam with PEG and PVP demonstrated comparable results [39, 42].

### 3.7. SEM

**Figure 7:**
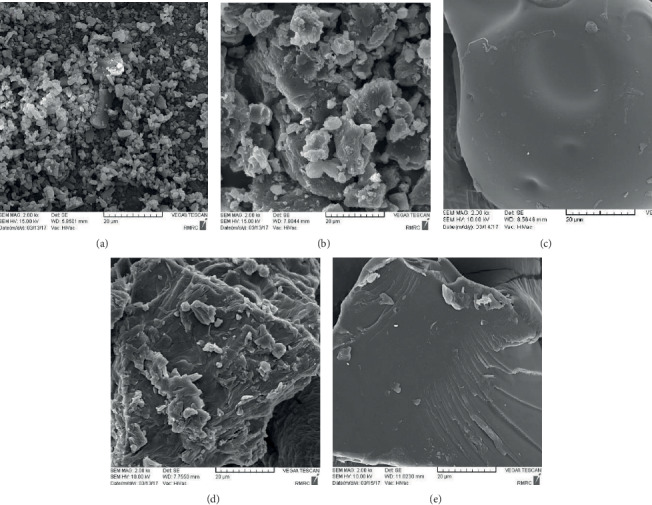
SEM images of (a) DFX powder, (b) PEG4000, (c) PVP K25, (d) drug/PEG4000 SD (1 : 1), and (e) drug/PVP K25 SD (1 : 1).

**Figure 8:**
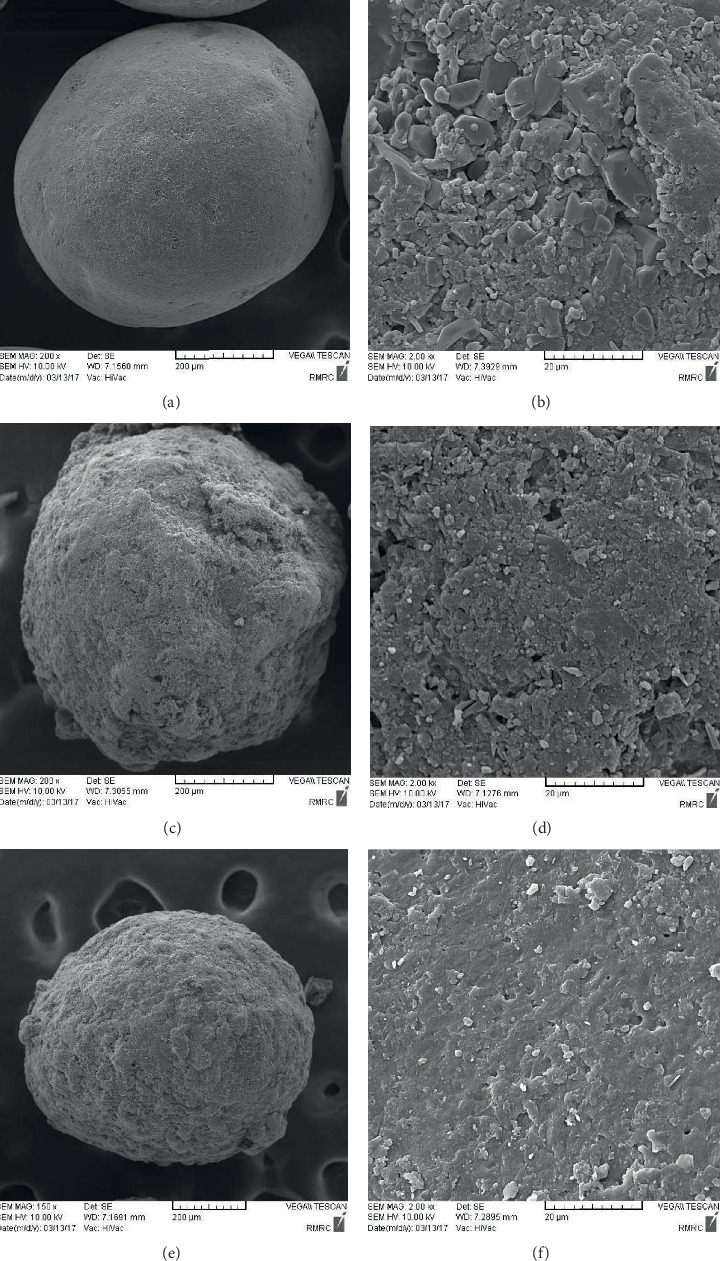
SEM photomicrographs of (a, b) a nonpareil seed, (c, d) DFX/PEG SD pellet, and (e, f) DFX/PVP SD pellet.

The SEM images of DFX powder, PVP K25, PEG4000, and the SDs are displayed in Figure 7. The untreated DFX powder consisted of a mixture of small and large crystals (Figure 7(a)). In contrast, electron micrographs of SDs did not exhibit the crystal form of DFX, and there was a major change in the morphology of the polymers (Figures 7(d) and 7(e)). As observed under a SEM, the morphologies of nonpareil seeds were spherical (Figure 8(a)). SDs were uniformly distributed on the surface of the nonpareil seeds; however, pellets coated by PVP SD showed a smoother surface than pellets coated by PEG SD.

### 3.8. XRPD

**Figure 9:**
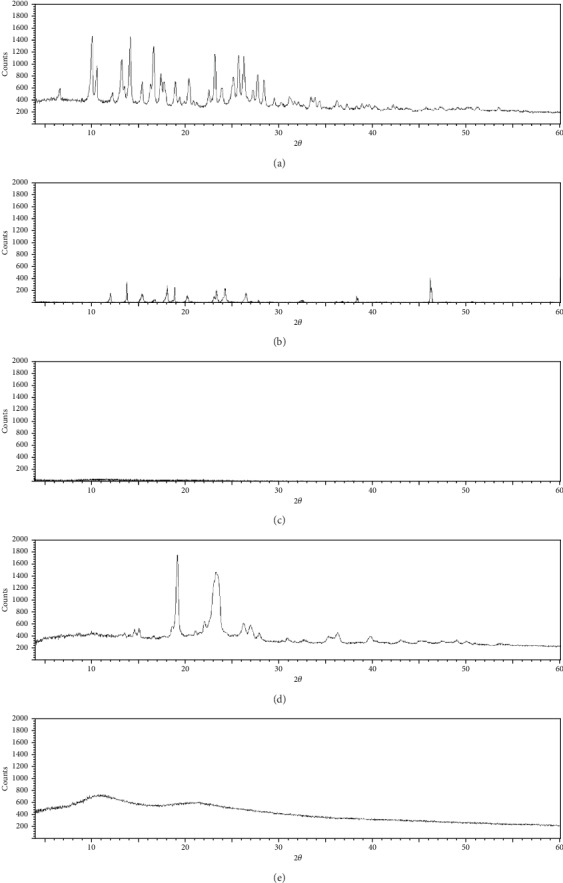
Powder X-ray diffractogram of (a) DFX powder, (b) PEG4000, (c) PVP K25, (d) drug/PEG4000 SD (1 : 1), and (e) drug/PVP K25 SD (1 : 1).

The crystallinity of DFX, the polymers, and the prepared SDs was investigated using XRPD. (Figure 9). The figure shows that DFX and PEG have crystalline structure as both of them show sharp peaks. The diffraction patterns of DFX indicated representative high-intensity diffraction peaks at 2*θ* values of 10.07°, 10.64°, 13.22°,14.15°, 16.66°, 23.25°, and 25.68°. It is obvious from XRD of SD formulations containing PEG 4000; still, some of the distinctive peaks of DFX are visible, which indicates that not all the drug in this formulation is in an amorphous state. In the case of SD formulation containing PVP, it is clear that all major characteristic crystalline peaks for the drug disappeared in the diffractogram (halo shape) which indicates the DFX is entirely in an amorphous state or molecularly spread in the matrix of polymer [43]. This data supported the data obtained by DSC and FTIR.

## 4. Conclusion

This report disclosed that the preparation of drug-polymer SD had a significant effect on the solubility and dissolution rate of DFX. SDs fabricated by PVP K25 showed more improvement in insolubility and *in vitro* drug release than those prepared by PEG4000. In addition, the results also revealed the feasibility of preparing acceptable SD-coated pellets by the powder layering technique. Up to 68.32% of the drug could be entrapped in the coating layer with PVP K25 SD. In conclusion, an effective multiparticulate dosage form can be developed to accelerate the delivery of poorly soluble drugs by spreading the drug in the hydrophilic polymer matrix and coating the layer of the mixture on inert pellet cores.

## Figures and Tables

**Table 1:** Solubility test of different SD formulations and free DFX after 24 hours (solubility data are shown as the mean ± standard deviation, *n* = 3).

Formulation number	Drug: polymer ratio	DFX (mg)	Solubility in 24 h (*μ*g/ml)
F1	PEG 1 : 1	50	50.78 ± 1.98
F2	PEG 1 : 3	50	56.73 ± 0.98
F3	PEG 1 : 5	50	62.81 ± 1.57
F4	PVP 1 : 1	50	74.95 ± 1.53
F5	PVP 1 : 3	50	85.64 ± 2.44
F6	PVP 1 : 5	50	95.73 ± 1.53
F7	Free DFX	50	38.02 ± 0.08

**Table 2:** Composition of pellet formulations amount of (all ingredients are reported as %w/w based on the weight of core material).

Formulation	DFX	Polymer		Lactose (filler)	Talc (anti-tacking agent)	Aerosil (glidant)
		PEG 4000	PVP K25			
F1	2	2	—	30	1	1
F2	2	6	—	26	1	1
F3	2	10	—	22	1	1
F4	2	—	2	30	1	1
F5	2	—	6	26	1	1
F6	2	—	10	22	1	1
F7	2	—	—	32	1	1

DFX: deferasirox; Na-CMC: sodium carboxymethyl cellulose; Aerosil: silicon dioxide.

**Table 3:** Dissolution characteristics of DFX SDs after 10 and 60 min in phosphate buffer at 37°C.

Formulation	%DE10	%DE60	%DP10	%DP60
F1	15.085	47.725	30.173	74.224
F2	16.162	55.761	32.325	88.385
F3	18.207	59.128	36.415	88.284
F4	21.775	60.27	43.55	84.763
F5	25.394	67.974	50.778	91.965
F6	28.455	73.12	56.917	96.523
F7	6.86	30.063	13.722	57.696

DE: dissolution efficiency; DP: dissolution percentage.

**Table 4:** *In vitro* release kinetic parameters.

	Zero-order (*R*^2^)	First-order (*R*^2^)	Korsmeyer-Peppas		Higuchi
			(*R*^2^)	*n*	(*R*^2^)
F1	0.839	0.724	0.966	0.465	0.936
F2	0.707	0.607	0.908	0.530	0.844
F3	0.698	0.656	0.899	0.540	0.837
F4	0.776	0.689	0.953	0.368	0.830
F5	0.665	0.695	0.899	0.344	0.747
F6	0.562	0.517	0.847	0.312	0.718
F7	0.917	0.778	0.977	0.737	0.976

## Data Availability

The data used to support the findings of this study are available from the corresponding author upon request.
